# The distance and median problems in the single-cut-or-join model with single-gene duplications

**DOI:** 10.1186/s13015-020-00169-y

**Published:** 2020-05-04

**Authors:** Aniket C. Mane, Manuel Lafond, Pedro C. Feijao, Cedric Chauve

**Affiliations:** 1 Department of Mathematics, Simon Fraser University, 8888 University Drive, Burnaby, V5A 1S6 Canada; 2 Department of Computer Science, Université de Sherbrooke, Boulevard de l’Université, Sherbrooke, J1K 2R1 Canada; 3 School of Computing Science, Simon Fraser University, 8888 University Drive, Burnaby, V5A1S6 Canada

**Keywords:** Genomes rearrangement, Gene duplication, Genomic distance, Genome median

## Abstract

**Background.:**

In the field of genome rearrangement algorithms, models accounting for gene duplication lead often to hard problems. For example, while computing the pairwise distance is tractable in most duplication-free models, the problem is NP-complete for most extensions of these models accounting for duplicated genes. Moreover, problems involving more than two genomes, such as the genome median and the Small Parsimony problem, are intractable for most duplication-free models, with some exceptions, for example the Single-Cut-or-Join (SCJ) model.

**Results.:**

We introduce a variant of the SCJ distance that accounts for duplicated genes, in the context of directed evolution from an ancestral genome to a descendant genome where orthology relations between ancestral genes and their descendant are known. Our model includes two duplication mechanisms: single-gene tandem duplication and the creation of single-gene circular chromosomes. We prove that in this model, computing the directed distance and a parsimonious evolutionary scenario in terms of SCJ and single-gene duplication events can be done in linear time. We also show that the directed median problem is tractable for this distance, while the rooted median problem, where we assume that one of the given genomes is ancestral to the median, is NP-complete. We also describe an Integer Linear Program for solving this problem. We evaluate the directed distance and rooted median algorithms on simulated data.

**Conclusion.:**

Our results provide a simple genome rearrangement model, extending the SCJ model to account for single-gene duplications, for which we prove a mix of tractability and hardness results. For the NP-complete rooted median problem, we design a simple Integer Linear Program. Our publicly available implementation of these algorithms for the directed distance and median problems allow to solve efficiently these problems on large instances.

## Background

Reconstructing the evolution of genomes at the level of large-scale genome rearrangements is an important problem in computational biology; e.g. [1, 2]. For a given genome rearrangement model, there are several computational problems that can be defined, from the computation of pairwise distances to the reconstruction of complete phylogenetic trees, often following a parsimony approach [3]. Among these problems, the reconstruction of ancestral gene orders given a species phylogeny has been considered in various frameworks, including the small parsimony problem (SPP), which aims at computing gene orders at the internal nodes of the given species phylogeny while minimizing the sum of the genome rearrangement distances along its branches. The simplest instance of the SPP is the Genome Median Problem, where the given species phylogeny contains a single ancestral node.

For most genome rearrangement models that do not consider gene duplication, computing the pairwise distance is tractable [3]. This contrasts with the median problem, that has been shown to be intractable in most models. The median problem was introduced in 1996 [4], motivated by its application in heuristics for the SPP [5]. Early results suggested that, even in the simple breakpoint distance model, computing a median gene order is intractable [6], and heuristics based on the Traveling Salesman Problem were introduced to solve the breakpoint median problem [5, 7]. However, in 2009, Tannier, Zheng and Sankoff proved that computing a median gene order that is allowed to contain an arbitrary mixture of linear and circular fragments is tractable in the breakpoint distance model, by using a reduction to a maximum weight matching (MWM) problem [8]. This tractability result, the first of its kind in genome rearrangement algorithms, renewed the interest in gene order median problems, although most of the following work presented intractability results, even on variations of the breakpoint distance [9–11]. A notable exception was the Single-Cut-or-Join (SCJ) distance, introduced by Feijão and Meidanis [12], where it was shown that both the median problem and the SPP are tractable.

Gene duplication is another important evolutionary mechanism; genes can be duplicated through different kinds of evolutionary events, from single-gene duplication to whole-genome duplications (WGD) [13, 14]. The first models of evolution by genome rearrangements considered the case of genomes with equal gene content, thus disregarding gene duplication and gene loss. However, for most models that account for gene duplication, the pairwise distance problem is intractable. For example, whereas the distance between two genomes can be computed in linear time for genomes without duplicated genes under the Double-Cut and Join (DCJ) model, it becomes NP-complete to compute the distance when duplicated genes are considered [15, 16], although it can be approximated when the gene content in both genomes is balanced [17]. So far, even in simpler genome rearrangement models, the general problem of computing a distance with duplicated genes is difficult [18, 19], with the exception of polynomial time algorithms for two extensions of the SCJ model that include large-scale duplications: the SCJ double distance [12], where duplicated genes occur through a WGD, and the SCJ and whole chromosome duplication (WCD) problem, motivated by cancer genomics [20].

In the present paper, we introduce novel results about the pairwise distance and median problems, in a model accounting for gene duplications. Our evolutionary model is an extension of the SCJ model that includes single-gene duplications of two different types, Tandem Duplications (TD) or Floating Duplication (FD) in which a new copy is introduced as a circular chromosome. We call this genome rearrangement model the SCJ-TD-FD model. The the pairwise distance problem we consider, is a *directed distance* problem, where we assume that one genome, say *A*, is duplication-free, while the other one, denoted by *D*, can contain duplicated genes. This setting is motivated by (1) the SPP where distances are considered along the branches of a given species phylogeny, so between an ancestral genome *A* and a descendant genome *D* and (2) the fact that developments in phylogenomics methods—especially gene trees/species tree reconciliation algorithms—make it realistic to assume that orthology relations between genes of an ancestral gene order and genes of a descendant gene order are known, allowing to see the ancestral gene order as duplication-free with regard to its descendant. This general framework was introduced by Sankoff and El-Mabrouk in [21] (see also [22]) and was later implemented in the $\hbox {DeCo}^*$ family of algorithms [23] to reconstruct ancestral gene adjacencies in a duplication-aware evolutionary model from data including extant gene orders and reconciled gene trees. We show that in the SCJ-TD-FD model the directed pairwise distance problem is tractable, and that a parsimonious scenario, can be computed in linear time. We also introduce two genome median problems, the directed median problem and the rooted median problem. In the directed median, we aim to reconstruct a parsimonious duplication-free ancestral gene order from the gene orders of $k\ge 2$ descendant genomes, that minimizes the sum of the directed distances to the *k* descendants gene orders. In the rooted median problem, we aim to reconstruct a parsimonious median genome between an ancestral genome *A* and $k\ge 2$ descendant genomes, where we assume that the gene content of the median is given and that unambiguous orthology relations between the median genes and the given gene orders are provided. We prove that the directed median problem is tractable, while the rooted median problem is NP-complete, and we provide a simple simple Integer Linear Program (ILP) for this problem, based on a reduction to a colored MWM problem. We evaluate our algorithms on simulated data and observe that they generate efficiently very accurate results.

## Preliminaries

### Genes, adjacencies and genomes

A genome consists of a set of chromosomes, each being a linear or circular ordered set of oriented genes. Following the usual encoding of gene orders, we represent a genome by its *gene extremity adjacencies*, which we call adjacencies from now. In this representation, a gene *g* is represented using a pair of gene extremities $(g_t, g_h)$, $g_t$ denotes the tail of the gene *g* and $g_h$ denotes its head, and an *adjacency* is a pair of gene extremities that are adjacent in a genome. If a gene $g_i$ is denoted with a subscript, we will denote the tail of $g_i$ by $g_{i,t}$ and its head by $g_{i,h}$. A gene extremity is *free*, also called a *telomere*, if it does not belong to an adjacency. A *chromosome* is a maximal contiguous sequence of genes; a chromosome with *k* genes can have either $k-1$ adjacencies, in which case it is a *linear* chromosome, or *k* adjacencies, in which case it is a *circular* chromosome.

In our work, we consider that genes can be duplicated. This implies that a given gene *g* can have multiple copies in a genome, the number of copies being called its *copy number*. Given a set of genomes, we call a *gene family* all copies of a given gene observed in the genomes. A set of genomes is said to have *equal gene family content* if every genome contains at least one gene from every gene family. A genome in which every gene has copy number 1 (i.e. is duplication-free) is called a *trivial genome*. A gene family is said to be trivial if each genome contains a single gene from this family.

It is important to note that a non-trivial genome can not always be represented unambiguously by its adjacencies, that can form a *multi-set*, unless we distinguish the copies of each gene, for example by denoting the copies of a gene *g* with copy number *k* by $g^1,\dots ,g^k$. Generally a multi-set of gene adjacencies can have several realizations as a gene order with duplicated genes. Nevertheless, in our work we identify a genome with its multi-set of adjacencies, as we will show this is sufficient in order to solve the directed pairwise distance problem we introduce.

**Fig. 1:**
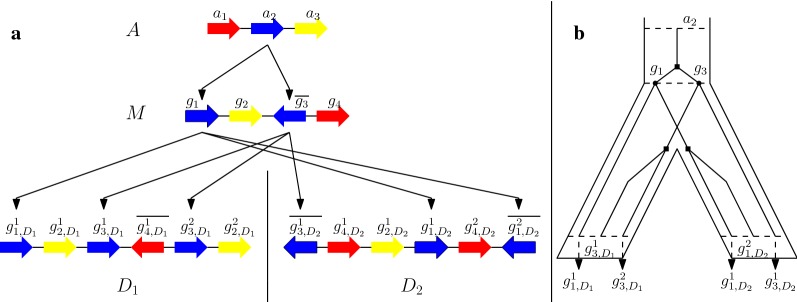
In **a** each color represents a gene family from *A*. Notice that each gene in *D*_1_ and $D_2$ can be traced to a unique gene in *M* whereas a gene from *A* might have multiple daughters in *M*. **b** displays the gene tree of the gene family in blue [indicated by arrows in (**a**)]. Since the gene $a_2$ undergoes duplication (dark squares) to form $g_1$ and $g_3$ in *M*, *M* is not trivial w.r.t *A*

For a given gene order *X*, we denote by $\Gamma _X$ its gene content, which is a set if *X* is trivial and a multi-set otherwise. We call the set induced by $\Gamma _X$ (which is exactly $\Gamma _X$ only if *X* is trivial) its *gene alphabet*; it follows that two gene orders *X* and *Y* have equal gene family content if and only if they have the same gene alphabet. A key assumption in our work is that when we compare a pair of gene orders, we assume that one, say *A* is an ancestor of the second one (say *D*). In that context, gene families define complete bipartite graphs (bi-cliques) between the two multi-sets of genes of *A* and *D*, that define *orthology relations*; we say that these orthology relations are *unambiguous* if all such bi-cliques contain a single gene of *A*, which is equivalent to state that all members of a gene family in *D* evolved, by gene duplications, from a unique gene in *A*. As a consequence, if a genome *A* is not trivial but is compared to a descendant genome *D* such that orthology relations between *A* and *D* are unambiguous, we say that *A* is *trivial with respect to D*. This is illustrated in Fig. 1. In a practical context, for a given set of extant gene orders and a species phylogeny for these gene orders, unambiguous orthology relations can be obtained, among other methods, by computing a gene tree per gene family and reconciling the gene trees with the species phylogeny; we refer to [22] for a discussion on the use of reconciled gene trees for the study of gene orders with duplicated genes.

Given two multi-sets *X* and *Y* of adjacencies, we define $X-Y$ as the multi-set obtained as follows: it contains *k* copies of a given adjacency if and only if *X* contains exactly *k* more occurrences of this adjacency than *Y*; so if *Y* contains more copies of an adjacency than *X*, $X-Y$ contains no copy of this adjacency. Note that this operator is not symmetric as $X-Y$ can be different from $Y-X$.

### Evolutionary model: the SCJF-TD-FD model

**Fig. 2:**
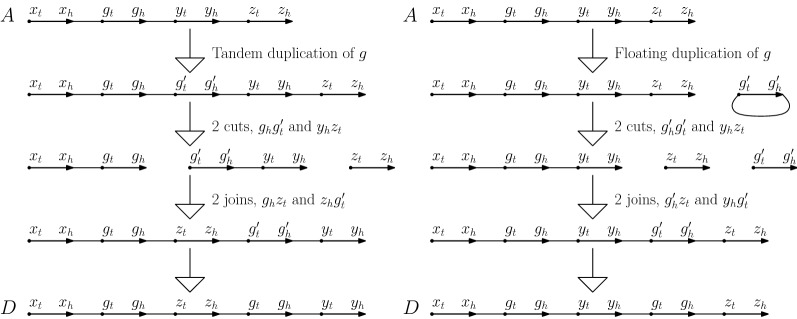
(Left) A tandem duplicate of gene *g* is introduced. The adjacency $g_hy_t$ has been replaced by $g'_hy_t$ and an adjacency $g_hg'_t$ has been introduced. In this case the total number of operations to obtain *D* from *A* is 5. Note that the number of cut and join operations is dependent on the adjacencies of the gene *g* in *A* and *D*. (Right) A floating duplicate of gene *g* is introduced. An adjacency $g'_hg'_t$ has been added. In this case the total number of operations to obtain *D* from *A* is 5

In the SCJ-TD-FD model, genome rearrangements are modeled by *Single-Cut-or-Join* (SCJ) operations, which either delete an adjacency from a genome (a cut) or join a pair of free gene extremities (a join), thus forming a new adjacency. For duplication events, we consider two types of duplications, both creating an extra copy of a single gene: *Tandem Duplications* (TD) and *Floating Duplications* (FD). A tandem duplication of an existing gene *g* introduces an extra copy of *g*, say $g'$, by adding an adjacency $g_h g'_t$, and, if there was an adjacency $g_h x$ by replacing it by the adjacency $g'_h x$. A floating duplication introduces an extra copy $g'$ of a gene *g* as a single-gene circular chromosome by adding the adjacency $g'_h g'_t$. We illustrate the two kinds of duplications in Fig. 2.

The motivation for considering floating duplications is that gene insertions and gene deletions have been modeled with artificial circular chromosomes before, greatly simplifying how to deal with such type of operations. For instance, in DCJ model, a deletion of a gene can be seen as a DCJ operation that applies two cuts to remove the given gene from a chromosome, followed by two joins to “repair” the broken chromosome and to circularize the deleted gene. A gene insertion is the inverse of this operation. This idea was effectively used in the DCJ-indel model by Compeau [24].

Note also that our model does not include gene loss. The extension to include this evolutionary mechanism will be developed in a further work.

### Problem statements

The first computational problem we consider is the directed SCJ-TD-FD (d-SCJ-TD-FD) distance problem. We consider a model of *directed evolution* in which, when comparing two genomes, we assume one, denoted by *A*, is an ancestor of the other genome, denoted by *D*; as the SCJ-TD-FD model does not consider gene losses, both *A* and *D* have equal gene family content. Moreover, we require that the genome *A* is trivial with respect to *D*. The d-SCJ-TD-FD distance problem asks to compute the minimum number of SCJ, TD and FD operations needed to transform the ancestral gene order *A* into the descendant gene order *D*, denoted by $d_{\text {DSCJ}}(A,D)$. Note that formally this way to measure the dissimilarity between genomes is not a distance as it is not symmetric, due to the assumption of *A* being trivial with respect to *D*; it is easy to see that symmetry is the only property of a distance which is not satisfied, so we are dealing actually with a *quasimetric*, although we call it a distance for the sake of consistency with the terminology used in standard genome rearrangement problems.

The second problem we consider is a genome median problem, the directed *SCJ-TD-FD (d-SCJ-TD-FD) median problem*. It is defined as follows: given $D_1,\dots ,D_k$ ($k \ge 2)$ (possibly) non-trivial genomes having equal gene family content, we want to compute a trivial genome *M* on the same set of gene families, that minimizes

1 $$\begin{aligned} \sum _{i=1}^k d_{\text {DSCJ}}(M,D_i). \end{aligned}$$

Note that, while generally in genome rearrangement models the median of two problem is trivial, as any gene order along a parsimonious scenario between $D_1$ and $D_2$ is a median, it is different in the context of a directed distance, which motivates this problem.

Last, we consider another genome median problem, motivated by the SPP on rooted phylogenies, the rooted *SCJ-TD-FD (r-SCJ-TD-FD) median problem*. It takes as input (1) $k+1\ge 3$ genomes, *A*, $D_1,\dots ,D_k$ with equal gene family content, such that *A* is a trivial genome, ancestor to the $D_i$s, (2) the gene content $\Gamma _M$ of a median genome *M* which is a descendant of *A* and an ancestor of the genomes $D_1,\dots ,D_k$, and (3) unambiguous orthology relations between $\Gamma _A$ and $\Gamma _M$, and between $\Gamma _M$ and the genes of each $D_i$ (so one set of orthology relations for each $D_i$). The goal of the rooted median problem is to compute a gene order for *M* (i.e. a set of adjacencies on $\Gamma _M$) minimizing

2 $$\begin{aligned} d_{\text {DSCJ}}(A,M)+\sum _{i=1}^k d_{\text {DSCJ}}(M,D_i). \end{aligned}$$

Note that in the objective function above, when *M* is compared to *A*, genes from the same family are indistinguishable, while they are distinguished when *M* is compared to each $D_i$ as the provided orthology relations between *M* and each $D_i$ are unambiguous. So $\Gamma _M$ can be considered as a multi-set in $d_{\text {DSCJ}}(A,M)$ and a set in the terms $d_{\text {DSCJ}}(M,D_i)$.

## The pairwise distance problem

In this section, we show that the d-SCJ-TD-FD distance between *A* and *D* can be calculated with the symmetric difference between the adjacency (multi)sets of the input genomes, with extra terms accounting for the observed TD and FD in *D*. We then provide an alternative formula for the distance, that is more amenable to being implemented in a Integer Linear Program (ILP) in the rooted genome median problem. Last, we describe a linear time algorithm to compute a parsimonious SCJ-TD-FD scenario.

### The directed SCJ-TD-FD distance

**Fig. 3:**
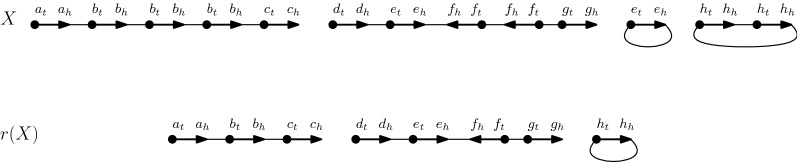
An example of the reduced genome *r*(*X*), of the genome *X*. Note that an instance of $h_hh_t$ is retained so that *r*(*X*) contains at least one representative of gene family *h*. All observed duplications are removed in *r*(*X*). Here, $t(X) = |X - r(X)| = 5$

Given a gene *g*, we call a *g*-*linear array* a sequence of consecutive adjacencies $g_hg_t$; if this sequence forms a circular chromosome, it is called a *g*-*circular array*. Given a genome *X*, we call an adjacency $g_hg_t$ an *observed duplication* if *g* has more than one copy in *X*. Observed duplications are part of a *g*-linear array or a *g*-circular array. Let *r*(*X*) be the genome obtained from *X* by successively deleting an observed duplication from *X*, chosen arbitrarily, until there remains no observed duplication. This corresponds to deleting every $g_hg_t$ adjacency, except that we keep one in the special case in which all copies of *g* are organized in *g*-circular arrays, as shown in Fig. 3. We call *r*(*X*) the *reduced* genome of *X*.

It is immediate to see that the order in which observed duplications are removed does not matter on the result, i.e. *r*(*X*) does not depend on this order. We define $t(X) = |X - r(X)|$, the number of adjacencies to delete to transform *X* into *r*(*X*). Last, for two genomes *X* and *Y*, we denote by $\delta (X,Y)$ the absolute difference between the number of genes of *X* and the number of genes of *Y*. Our first main result is given in Theorem 1, which generalizes the SCJ distance formula introduced in [12] in the case where *A* and *D* are both trivial and with equal gene content.

#### **Theorem 1**

*Let A and D be two gene orders with equal gene family content, such thatA is trivial with respect to D*.

3 $$\begin{aligned} d_{\text {DSCJ}}(A,D) = | A - r(D) | + | r(D) - A | + 2\delta (A,r(D)) + t(D). \end{aligned}$$

We provide in Additional file 1 a complete proof that we outline here. We first need to define the notion of *context conservation* of a gene between *A* and *D*. Assume that a gene *g* in *A* is not a telomere (and so there are two adjacencies involving *g* in *A*, say $g_t x$ and $g_h y$) and there is a copy of *g* in *D* whose extremities form also adjacencies $g_t x$ and $g_h y$. We say that the context of *g* is *strongly conserved* between *A* and *D*. Note that *x* and *y* do not need to belong to trivial gene families and there might be several copies of *x*, *y*, *g* in *D* that conserve the context of *g* in *A*. Assume now that the context of *g* is not strongly conserved between *A* and *D*, but both adjacencies involving *g*, $g_t x$ and $g_h y$, are present in *Dusing different copies* of *g*. We say that the context of *g* is *weakly conserved* between *A* and *D*. Again *x* and *y* need not to be trivial gene families and there might be several occurrences of adjacencies $g_t x$ and $g_h y$ in *D*. Last, if the context of *g* in *A* is neither strongly nor weakly conserved, and so at most one adjacency involving *g* in *A* is also present in *D*, then we say the context of *g* is *not conserved*. The principle of the proof is to proceed by induction on the number of duplicate copies in *r*(*D*) (which is equal to $\delta (A,r(D))$) and to pick an arbitrary gene from a non-trivial gene family for which one duplicate is introduced using an FD if the context is strongly conserved, a *TD* if it is weakly conserved and either an FD or a TD (both can be chosen arbitrarily) if the context is not conserved.

We now introduce an alternative formula to compute the directed distance, easier to implement in an ILP than the formula provided in Theorem 1 as it does not require to consider the reduction *r*(*D*) of *D*. To rewrite $d_{\text {DSCJ}}(A, D)$, we introduce an indicator variable $\alpha _{g,AD}$, where $\alpha _{g,AD} = 1$ if $g_hg_t$ is an adjacency present in both *A* and *D*, but all its occurrences in *D* were removed while reducing *D*. Formally, $\alpha _{g,AD} = 1$ if $g_hg_t \in A \cap D$ and $g_hg_t \notin r(D)$; otherwise $\alpha _{g,AD} = 0$. We then obtain the following result, whose proof is also provided in Additional file 1.

#### **Corollary 2**

Let *A* and *D* be two gene orders with equal gene family content, such that *A* is trivial with respect to *D*.

4 $$\begin{aligned} d_{\text {DSCJ}}(A, B) = |A - D| + |D - A| + 2\delta (A, D) - 2t(D) + 2\sum _{g \in \Gamma _A}\alpha _{g,AD} \end{aligned}$$

### Computing a parsimonious scenario

It follows from Theorem 1 that computing the d-SCJ-TD-FD distance can be done in linear time in the size of the considered genomes *A* and *D*. However, unlike in the case where both *A* and *D* are trivial, this does not imply in a straightforward way an algorithm to transform *A* into *D*, due to the fact that an adjacency multi-set can have several realizations. Nevertheless, we present a simple algorithm that computes a parsimonious scenario in terms of duplications, cuts and joins from *A* to *D*, Algorithm 1. 
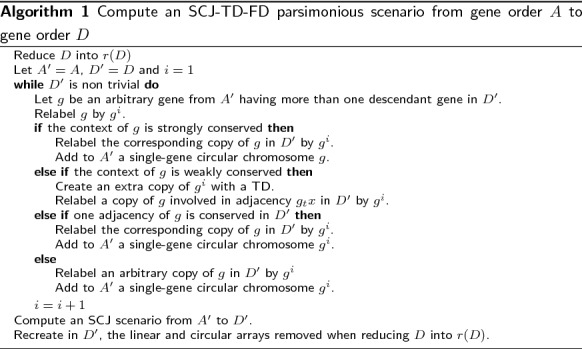


#### **Theorem 3**

*Let Aand Dbe two gene orders with equal gene family content, such that Ais trivial with respect to Dand D has*$n_D$*genes. Algorithm 1 computes a parsimonious SCJ-TD-FD scenario that transforms Ainto D and can be implemented to run in time and space*$O(n_D)$.

The correctness of the algorithm follows immediately from the fact that it implements exactly the rules described to compute the SCJ-TD-FD distance (proof of Theorem 1). The linear time and space complexity follows from the fact that these rules are purely local and require only to check for the conservation of adjacencies in both considered genomes. Every iteration of the while loop in Algorithm 1 takes place only if there is a non-trivial gene family left in $D'$. The maximum number of iterations is the number of duplicates genes, $n_d = n_D - n_A$ which is $O(n_D)$ when $n_D \ge n_A$. In each iteration, we check if the context of the chosen gene *g* is strongly conserved, weakly conserved or not conserved. This involves trying to match the adjacencies of *g* in *A* with those in the adjacency set of $D'$ that involve a copy of *g*. This can be done in constant time, with a linear time preprocessing of the data. Hence, the worst-case time complexity is $O(n_D)$.

## The directed and rooted median problems

### The directed median problem

Let us remind that the *directed median problem* asks, given *k* non-trivial genomes $D_1, \dots , D_k$, $k \ge 2$, with equal gene family content, to find a trivial genome *M*, such that $\sum _{i=1}^k d_{\text {DSCJ}}(M,D_i)$ is minimized. We denote by $\Gamma _M$ the gene content of *M*, that is the set induced by the multi-sets $\Gamma _{D_i}$. We denote by *n* the total number of adjacencies in the $D_i$s, $n=\sum _{i=1}^k|D_i|$ and by *m* the total number of gene families, i.e. $m=|\Gamma _M|$.

We first assume that the genomes $D_1,\dots ,D_k$ are reduced. We define the *scores*(*M*) of a trivial genome *M* as

$$\begin{aligned} s(M) = \sum _{i=1}^k d_{\text {DSCJ}}(M,D_i) = \sum _{i=1}^k \left( |M-D_i| + |D_i - M| + 2\delta (M,D_i) \right) \end{aligned}$$

Using the identity $|M-D| + |D-M| = |M| + |D| - 2|M \cap D|$, that holds even if the *D* is a multi-set, and denoting $N_d = \sum _{i=1}^k\left( 2\delta (M,D_i) + |D_i|\right)$, we derive

$$\begin{aligned} s(M) = N_d - \left( 2\sum _{i=1}^k | M \cap D_i| - k |M| \right) . \end{aligned}$$

Therefore, minimizing *s*(*M*) is equivalent to maximizing $2\sum _{i=1}^k | M \cap D_i| - k |M|$.

For a given adjacency *a*, let $\gamma _i(a)$ be 1 if $a \in D_i$, and 0 otherwise. The score of a genome with a single adjacency *a* is $s(\{a\}) = N_d - \left( 2\sum _{i=1}^k \gamma _i(a) - k\right)$. This motivates the following approach, similar to the breakpoint median algorithm of [8]. We build a graph *G* where the vertices are the extremities (head and tail) of the genes of $\Gamma _M$, and weighted edges are defined as follows: for any edge $e=(x,y)$ the weight of *e* is $w(e) = 2\sum _{i=1}^k \gamma _i(e) - k$. So any edge that does not appear in at least half of the descendant genomes has a negative weight. Any matching on *G* defines a trivial genome, having the adjacencies corresponding to the edges in the matching; so from now we identify matchings in *G* and trivial genomes on $\Gamma _M$. We denote the weight of *M* as $w(M)=\sum _{e \in M}w(e)$. It follows that

$$\begin{aligned} \begin{aligned} s(M)&= N_d - \sum _{e \in M} \left( 2\sum _{i=1}^k \gamma _i(e) - k\right) = N_d - w(M). \end{aligned} \end{aligned}$$

Therefore, solving the MWM problem on *G* solves the d-SCJ-TD-FD median problem.

To handle the case where some $D_i$ are not reduced, we rely on the fact that the genomes can be reduced first without impacting the optimality of a trivial genome obtained by a MWM. Combined with the tractability of computing a MWM [25], and the fact that any edge that does not correspond to an adjacency observed in a $D_i$ has negative weight and thus does not contribute to a MWM, we obtain the following result.

#### **Theorem 4**

*Let*$k\ge 2$* and*$D_1,\dots ,D_k$*be kgenomes having equal gene family content. Let mbe the number of gene families and n the number of adjacencies the*$D_i$*s. The directed SCJ-TD-FD median problem with input*$D_1,\dots ,D_k$* can be solved in time and space*$O(mn\log _2(m))$.

### The rooted median problem is intractable

We now describe two results for the rooted SCJ-TD-FD median problem. We remind that this problem asks, given $k+1$ non-trivial genomes $A, D_1, \dots , D_k$, $k \ge 2$, with equal gene family content, the gene content $\Gamma _M$ of a median genome and unambiguous orthology relations between $\Gamma _A$ and $\Gamma _M$ and $\Gamma _M$ and the $\Gamma _{D_i}$s, to find a gene order *M* on $\Gamma _M$, such that $d_{\text {DSCJ}}(A,M)+\sum _{i=1}^k d_{\text {DSCJ}}(M,D_i)$ is minimized. As *M* might be non-trivial with respect to *A* but is trivial with respect to the $D_i$s, we denote its adjacencies by $M_a$ in the former case ($M_a$ might be a multi-set of adjacencies) and *M* in the later (a set of adjacencies); so *M* is induced by distinguishing the copies of a same gene family in $M_a$. For a given adjacency *xy* on $\Gamma _M$, we denote by *a*(*x*)*a*(*y*) the adjacency on $\Gamma _A$ obtained by replacing *x* and *y* by their respective orthologs in *A*, denoted by *a*(*x*) and *a*(*y*).

Our first result is that, unlike the directed SCJ-TD-FD median problem, the rooted SCJ-TD-FD median problem is NP-complete. Our second result is a simple ILP to solve the rooted SCJ-TD-FD median problem.

#### **Theorem 5**

*The rooted SCJ-TD-FD median problem is NP-complete*.

The full proof of this result is given in Additional file 1. We provide here an outline of the proof, together with some comments on the specific instances for which the rooted median problem is shown to be intractable. We show that finding the optimal gene order for *M* is NP-complete even for $k=2$, by reduction from the 2P2N-3SAT problem [26] (This problem is sometimes called the (3,B2)-SAT problem, where B2 indicates that the literals are *balanced* with two occurrences each). In 2P2N-3SAT, we are given *n* variables $x_1, \ldots , x_n$ and *m* clauses $C_1, \ldots , C_m$, each containing exactly 3 literals. Each variable $x_i$ appears as a positive literal in exactly 2 clauses, and as a negative literal in exactly 2 clauses. Note that since each variable occurs in exactly 4 clauses and each clause has 3 literals, $m = 4n/3$. Then we show that from a given instance of 2P2N-3SAT, we can design a polynomial size instance of the r-SCJ-TD-FD median such that the initial 2P2N-3SAT instance is satisfiable if and only if there exists a median genome *M* satisfying

$$\begin{aligned} d_{\text {DSCJ}}(A, M_a) + d_{\text {DSCJ}}(M, D_1) + d_{\text {DSCJ}}(M, D_2) \le 2|D_1| - 2n + 4\delta (M, D_1). \end{aligned}$$

We can make two interesting observations about our hardness proof:

- In our reduction from 2P2N-3SAT, none of the considered genomes contain a *g*-tandem array or a *g*-chromosome. So our result also implies the hardness of the rooted median problem where the distance between two genomes *A* and *D*, where *A* is an ancestor of *D*, is computed in a simpler way as $|A-D|+|D-A|+2\delta (A,D)$, i.e. does not contain a term related to *r*(*D*).
- The reduction we provide involves $k=2$ and two descendant genomes $D_1,\ D_2$ such that $D_1=D_2$. It is somewhat striking to remark that computing the distance between *A* and *D* is tractable, while computing the distance between *A* and two identical copies of *D*, constrained by the gene content and orthology relations of an intermediate genome is hard. However our hardness proof does not imply that computing a median between *A* and $D_1$ (with given gene content and unambiguous orthology relations with *A* and $D_1$), and we even conjecture it is tractable.

### An integer linear program for the rooted median problem

We now describe a simple Integer Linear Program (ILP) to solve the rooted median problem. The key idea is again to convert the rooted median problem into an instance of a MWM problem, albeit with certain additional constraints. More precisely, in this approach we define a complete graph *G* on the extremities $g_h$ and $g_t$ of every gene *g* in $\Gamma _M$. A pair of distinct extremities defines an edge and thus a potential adjacency in *M*, which is thus defined by a matching in *G*. Each edge is assigned a weight that reflects the number of descendant genomes which contain the corresponding adjacency. Further, each edge is assigned a color that reflects its corresponding adjacency in the ancestral genome *A*, if any, and the number of colors of the selected edges also contributes to the weight of the matching defining the median *M*.

We first use Eq. (4) to reformulate the objective function of the rooted median problem. The claim below is formally proved in Additional file 1.

#### **Claim 1**

Minimizing the function Eq. (2) defining the evolutionary cost of a median *M* is equivalent to maximizing the following expression:

5 $$\begin{aligned} \sum _{i = 1}^{k} {\left( 2|M \cap D_i| - 2\sum _{g \in \Gamma _M}\alpha _{g,MD_i} \right) } + 2|A\cap M_a| + 2t(M_a) - 2\sum _{g \in \Gamma _A}\alpha _{g,AM_a} -(k+1)|M|. \end{aligned}$$

*An interpretation as a colored MWM problem.* In order to compute $|M \cap D_i|$, we use again variables $\gamma _i(e)$ as defined for the directed median algorithm, define a graph *G* over the vertex set $\Gamma _M$ and weighted edges, with a weight defined as $w(e) = 2\sum _{i = 1}^{k} {\gamma _i(e)} - (k+1).$

Since *M* is trivial with respect to every $D_i$, the weights for edges $e\in M$ in the graph *G* defined as in the directed median problem will account for the term $\sum _{i = 1}^{k} {2|M \cap D_i|} - (k+1)|M|$ in Eq. (5). However, this principle does not work with *A*. Indeed, it is possible that $x_1y_1 \in M$ and $x_2y_2 \in M$ such that $a(x_1)a(y_1) = a(x_2)a(y_2) \in A$. In this situation, only one of $x_1y_1$ or $x_2y_2$ can contribute to $|A \cap M_a|$, but both $|x_1y_1 \cap A|$ and $|x_2y_2 \cap A|$ are equal to 1. In other words, we cannot simply sum the adjacencies of $M_a$ which are present in *A*.

**Fig. 4:**
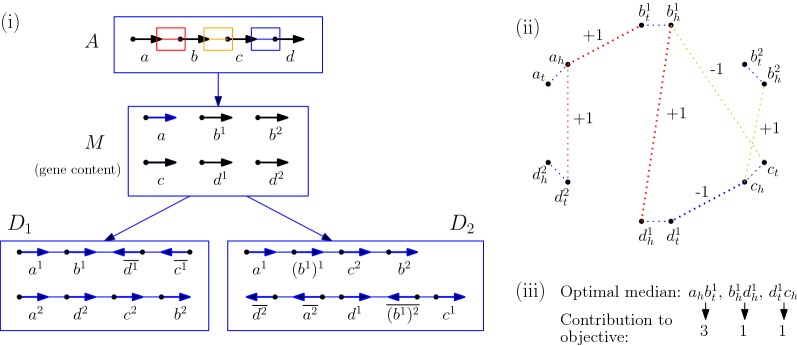
Part (i) of the figure shows an instance of the rooted median problem. Genomes *A*, $D_1$ and $D_2$ and the gene content of *M* are provided. Each adjacency in *A* has been assigned a *color family*. For instance, any adjacency $a_hb_t$ in the median belongs to the red family. Part (ii) translates the problem into an instance of the colored MWM problem. Only, candidate adjacencies, appearing in at least two out of the three given genomes can be seen in the graph. The bold edges, forming a matching on the graph, denote the optimal median. Part (iii) lists the contributions of each of the selected edges

To address this issue, we introduce the notion of a *color family* (see Fig. 4). Let $m_A$ be the number of adjacencies in *A*. Each number from the set $\{1,2,\ldots ,m_A\}$ represents a distinct color. We arbitrarily assign a distinct color to each adjacency in *A*. If *E*(*G*) is the edge set of *G*, representing all possible adjacencies in *M*, then every adjacency in *E*(*G*) is assigned a color from $\{1,2,\ldots ,m_A\} \cup \{0\}$, consistent with the orthology relations: the adjacency $xy \in M$ receives color $i \ne 0$ if the adjacency *a*(*x*)*a*(*y*) is present in *A* and is assigned color *i*, and color 0 if *a*(*x*)*a*(*y*) is not present in *A*. The set of adjacencies having the same color *i* form a color family, represented by $E_i$. We denote by *C* the coloring function $E(G) \rightarrow \{0, 1,\ldots , m_A\}$ defined as described above. Note that a color *i* contributes exactly once to the term $|A \cap M_a|$ if there exists at least one adjacency in *M* that belongs to the color family $E_i$.

*Candidate adjacencies.* With the aim to reduce the number of edges in the graph to be considered, we show that we only need to consider specific adjacencies, which we call *candidate adjacencies*. An adjacency *xy* is a *candidate adjacency* for the median *M* if at least $\left\lfloor \frac{k+1}{2} \right\rfloor + 1$ genomes from the set $\{A, D_1, D_2,\ldots , D_k\}$ contain *xy* (where here *A* contains *xy* if $a(x)a(y) \in A$). Lemma 6 below is proved in Additional file 1 and implies that the number of adjacencies to consider in our ILP is linear in the sum of the sizes of the input genomes.

#### **Lemma 6**

*There exists an optimal median consisting of only candidate adjacencies. Furthermore, when k is even, an adjacency which is not a candidate adjacency can not be a part of any optimal median.*

Let us remark that, as a consequence, the hardness of the rooted median problem stems from the fact that duplication from *M* to the $D_i$s can create conflicting adjacencies, where a median gene extremity belongs to several candidate adjacencies. It is interesting to observe that this can happen only due to convergent evolution, i.e. the fact that the same adjacency is created independently in several $D_i$s. This suggests that in the practical context of a limited level of convergent evolution, the rooted median problem is actually easy to solve with the need to rely on an ILP.

*An ILP for the rooted median problem.* We can now provide the complete ILP formulation to solve the rooted SCJ-TD-FD median problem. Let *x*(*e*) be the binary decision variable denoting the inclusion of edge (candidate adjacency) $e \in E(G)$ in *M*. Let *w*(*e*) be the weight of the corresponding edge. Also, let $c_i$ be the binary decision variable indicating the existence of at least one edge from color family $E_i$ in the median *M*. From the previous paragraph, one can write the objective function as

**Maximize**:

$$\begin{aligned} \sum _{e \in E(G)} {w(e)x(e)} + 2\sum _{i=1}^{m_A}{c_i} + 2t(M_a) - 2\sum _{g \in \Gamma _A} \alpha _{g,AM_a} - 2\sum _{i=1}^{k}\sum _{g \in \Gamma _M} \alpha _{g,MD_i} \end{aligned}$$

We now describe the constraints of the ILP. The first set of constraints concern the *consistency* of the set of chosen adjacencies, that ensures that each gene extremity in *M* belongs to at most one adjacency, or in other words that *M* is a matching for the graph *G* (these are the first two sets of constraints below). Next, we use an additional set of constraints to determine the values of $c_i$, $i = \{1,2,\ldots ,m_A\}$. If at least one adjacency of color *i* is present in the median, $c_i = 1$, otherwise $c_i=0$. The following inequalities define these color constraints:

6 $$\begin{aligned} \sum _{e=(y_h,z)}{x(e)}&\le 1&\forall y \in \Gamma _M \end{aligned}$$

7 $$\begin{aligned} \sum _{e=(y_t,z)}{x(e)}&\le 1&\forall y \in \Gamma _M\end{aligned}$$

8 $$\begin{aligned} c_i&= \left\lceil \frac{\sum _{C(e)=i}{x(e)}}{|E_i|} \right\rceil&\forall i \in \{1,2,\ldots ,m_A\} \end{aligned}$$

Note that for $c_i$ above, the constraints of the type $x = \lceil y \rceil$ are not linear, but if *x* is restricted to be in $\{0,1\}$, it can be replaced by the constraint $y \le x \le y + \epsilon$, where $\epsilon$ is very close to 1, say 0.999. A similar trick can be used for floor functions.

In order to compute $\alpha _{g,uv}$ for every pair (*u*, *v*)—where either $u=A,\ v=M_a$ or $u=M, v=D_i$ for some *i*—and every gene $g \in \Gamma _u$, we use some additional constraints. Let $p_v(e)$ be the binary variable denoting if the adjacency *e* exists in *v*. We use an indicator variable $\lambda _{g,uv}$ such that $\lambda _{g,uv} = 1$ if and only if all copies of *g* are involved in $g_hg_t$ adjacencies. Consequently, $\lambda _{g,uv} = 1$ ensures the existence of the $g_hg_t$ adjacency in *r*(*v*). Thus, $\lambda _{g,uv} = \left\lfloor \frac{n_v(g_hg_t)}{n_v(g)} \right\rfloor$. Further, we use $\Lambda _{g,uv}$ to indicate if at least one instance of $g_hg_t$ has been observed in *v*. Thus, we can represent $\Lambda _{g,uv}$ as $\left\lceil \frac{n_v(g_hg_t)}{n_v(g)} \right\rceil$. Note here that $n_v(g_hg_t)$ counts the occurrences of $g_hg_t$ adjacencies in *v* while $n_v(g)$ counts the number of copies of *g* in *v*. Since we already know the gene orders of *A* and each $D_i$, the values of $p_A(e)$ and $p_{D_i}(e)$ are known. Further, $p_M(e)=x(e)$. Thus, we obtain the following constraints for every gene *g* and branch (*u*, *v*):

9 $$\begin{aligned} \lambda _{g,uv}&= \left\lfloor \frac{n_v(g_hg_t)}{n_v(g)} \right\rfloor \end{aligned}$$

10 $$\begin{aligned} \Lambda _{g,uv}&= \left\lceil \frac{n_v(g_hg_t)}{n_v(g)} \right\rceil \end{aligned}$$

11 $$\begin{aligned} \alpha _{g,uv}&= \min (p_u(g_hg_t),\Lambda _{g,uv} - \lambda _{g,uv}) \end{aligned}$$

12 $$\begin{aligned} t_v(g)&= n_v(g_hg_t) - \lambda _{g,uv} \end{aligned}$$

We use the fact that if $g_hg_t \notin v$ for some *g* then $g_hg_t \notin r(v)$. Thus, if $g_hg_t \notin v$, $\lambda _{g,uv} = 0$ thereby ensuring the correctness of constraints to find $\alpha _{g,uv}$. Again, note that the $\min$ function is not linear, but that a constraint $x = \min (y,z)$ can be replaced by $x \ge y$ and $x \ge z$, assuming that $x,y,z \in \{0,1\}$.

Thus, there are $|m_M|$ binary variables *x*(*e*) where $|m_M|$ is the number of candidate adjacencies for the median. Additionally, there are $|m_A|$ binary variables, to account for the color of each adjacency in *A*. Further, for every gene *g* from $\Gamma _A$ or from $\Gamma _M$, there are 7 variables each, used in Eqs. (9–12). All together, there are $2|m_M|$ constraints pertaining to existence of median adjacencies, $|m_A|$ constraints to determine the inclusion of the color of each ancestral adjacency and finally $4(|\Gamma _A| + k|\Gamma _M|)$ constraints from (9–12).

## Experiments

We now describe two sets of experiments on simulated data. In the first one, we evaluate the ability of the directed distance to correctly estimate the number of evolutionary events when gene duplications occur through *segmental duplications* (a more realistic model than single-gene duplications). In the second set of experiments, we evaluate the ability of the rooted median to reconstruct an accurate ancestral gene order, again in a model where gene duplication is not restricted to single-gene duplications. We also describe the results of the rooted median ILP on real mosquito genomes data.

### The pairwise distance

We ran experiments on simulated instances with the aim to evaluate the ability of the d-SCJ-TD-FD distance to correlate with the true number of syntenic events. We followed a simulation protocol inspired from [15]. The code itself was programmed in Python and is available via github.^1^ We first describe the simulation protocol, followed by the results we obtained.

We started from a genome *A* composed of a single linear chromosome containing 1000 single-copy genes. Then, we transformed genome *A* into a genome *D* through a sequence of random segmental duplications and inversions. We fixed the number *N* of evolutionary events (from 50 to 500 by steps of 50) and the probability *P* that a given event is a segmental duplication (from 0 to 0.5 by steps of 0.1). A segmental duplication is defined by three parameters: the position of the first gene of the duplicated segment, the length of the duplicated segment, and the breakpoint where the duplicated segment is transposed into; we considered two models of segmental duplications, one with fixed segment length *L* (with *L* taking values in $\{1,2,5\}$) and one where for each segment, *L* is picked randomly (under the uniform distribution) in $\{1,2,5,10\}$. The breakpoints for inserting duplicated segments as well as inversions were chosen randomly, again under the uniform distribution. For each array of parameters, we ran 50 replicates.

**Fig. 5:**
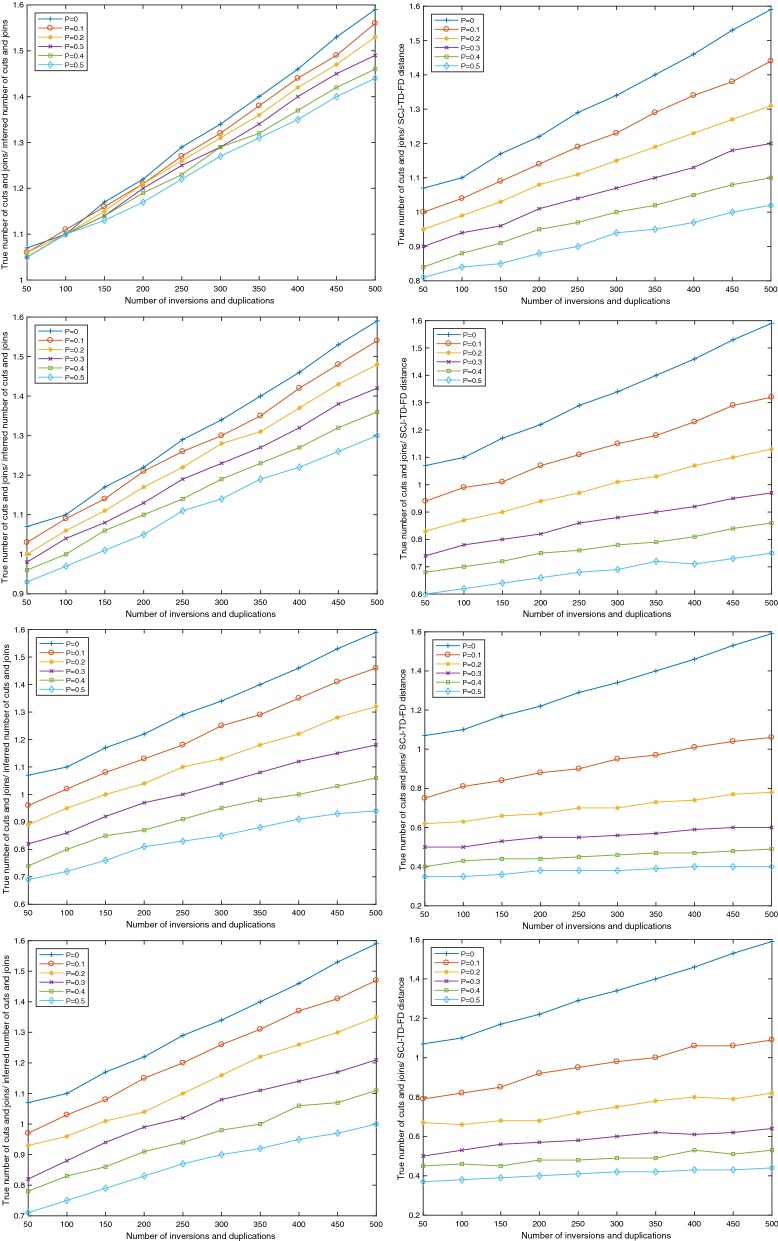
Experimental results, for four duplications parameters—single-gene segmental duplication (top row), two-genes segmental duplication (second row), five-genes segmental duplications (third row), variable length segmental duplications (bottom row)—and two measured quantities—inferred cuts and joins (left column) and SCJ-TD-FD distance (right column)

For each instance, we compared two quantities to the true number of cuts and joins in the scenario transforming *A* into *D*, which is roughly four times the number of inversions plus three times the number of segmental duplications: first we compared the full SCJ-TD-FD distance, defined as stated in Theorem 1 and the number of cuts and joins ($|A-D|+|D-A|$). Figure 5 illustrates the results we obtained.

We can make several observations from these results. The first one is a general trend that both measured quantities (the number of cuts and joins and the full SCJ-TD-FD distances) scale linearly with the true number of cuts and joins. The second observation is that, as expected, the slope and *y*-intercept of the graphs depend from both the frequency of duplications and the length of the duplicated segments. This leaves open the question of using the SCJ-TD-FD distance as an estimator of the number of cuts and joins in an evolutionary model where the probability of duplication compared to rearrangements (that can be estimated for example from reconciled gene trees and adjacency forests [23]) is given and the length of duplicated segments is expected to follow a well defined distribution.

### The rooted median problem

Next, we ran experiments on simulated data in order to evaluate the ability of the ILP to correctly predict the gene order of the median genome. The input for the program, including gene orders for the ancestor genome *A* and the descendant genomes $D_i$, along with the orthology relations, generated using the ZOMBI genome simulator [27]. The ILP was solved using the Gurobi solver.

*Simulations parameters.* Our input genomes consisted of one ancestor *A* and two descendants $D_1$ and $D_2$. We started with the ancestral genome *A* as a single circular chromosome consisting of 1000 genes, belonging to different gene families (so without duplicate genes). The genome *A* evolved into the median genome *M* using duplications, inversions and translocations. The genome *M* was further evolved along two independent branches to yield the descendant genomes, $D_1$ and $D_2$. The total number of rearrangements (inversions + translocations) from *A* to *M* and from *M* to $D_i$ was varied from 100 to 500, in steps of 100. The parameter for duplication events was kept constant throughout the experiments. The average number of duplicated genes, over all three branches collectively, was found to be 362.8 with a standard deviation of 82 genes. Considering the number of duplication events, the mean and standard deviation of segmental duplications over the three branches was 72.6 and 15.8 respectively. The lengths of segmental duplications, inversions and translocations were controlled using specific extension rates. These extension rates (all between 0 and 1) are the parameters of a geometric distribution dictating the respective lengths. Thus, the length of the segment being acted upon would be 1 if the extension rate parameter is set to 1 and would increase as the parameter value reduces. In our experiments, the inversion, translocation and duplication extension rates were 0.05, 0.3 and 0.2 respectively. For each setting (number of rearrangements) we ran 40 simulations.

*Results.* For each simulation, we compared the optimal median according to the ILP to the actual median generated by the simulator. For each group, we measured the average precision and recall statistics. The ILP predicts the median genome in the form of its adjacency set. Thus, in this context, precision refers to the ratio of number of correctly predicted adjacencies to the total number of adjacencies in the computed optimal median. On the other hand, recall represents the ratio of the correctly predicted adjacencies to the total number of adjacencies in the actual median. For each instance, we measured the number of candidate adjacencies used in the ILP. Additionally, to evaluate the effectiveness of our approach, we also measured the number of adjacencies in the solution which were common to all genomes ($A, D_1$ and $D_2$) and those common to only two of the three. An overview of the results is given in Table 1.

**Table 1:** Statistics of the ILP median experiment on simulated data

Events	Adj. in true median	Cand. adj.	Adj. in ILP median	Precision	Recall	$\%$ Adj. common to all genomes	$\%$ Adj. common to two genomes	No. of optimal solutions	Avg. time per run (in sec)
100	1514	1503	1493	0.9998	0.9859	86.43	13.57	2.3	53
200	1107	1062	1044	0.9991	0.9428	69.49	30.51	15.8	29
300	1312	1192	1155	0.9985	0.8758	52.94	47.06	40.3	38
400	1151	985	961	0.9981	0.8329	49.44	50.56	393.7	51
500	1430	1174	1132	0.9972	0.7897	46.68	53.32	3682.6	84

The ILP rarely predicts an erroneous adjacency to be a part of the optimal median, with a near-perfect precision. This property is observed throughout the experiments irrespective of the number of rearrangement events. On the other hand, the ILP predicts more than 90$\%$ of the median for lower rates of rearrangement and a decreasing trend is observed as the number of rearrangement events increase. This can be partly attributed to the decrease in the number of candidate adjacencies. In general, the number of candidate adjacencies is lower than the true number of adjacencies in the median, as including other adjacencies may result in a non-optimal median. This, however, emphasizes the practicality of Lemma 6, as the number of adjacency variables is significantly reduced. It can also be observed that the number of adjacencies common to all genomes decreases with increase in rearrangements. These adjacencies will be preferred by the ILP on account of higher weight.

Another notable observation is the increase in the number of optimal solutions with larger rates of rearrangement. This correlates naturally with the decrease in the number of adjacencies which are common to all genomes. For only 100 rearrangements, the ILP outputs a unique optimal median in most runs, with an overall average of 2.3 solutions. However, the average number of optimal solutions exceeded 3000 in case of 500 rearrangements. Despite a pool of optimal solutions, the SCJ distance between the actual median and an optimal median does not vary by much. If the SCJ distance between the actual median and a randomly chosen optimal median is *D*, then the distance between the actual median and any other optimal median was observed to stay within the range $(D-2, D+2)$. For most of our simulations, the ILP output an optimal median in under a minute, with the exception of the case with 500 rearrangement events.

## Conclusions

In this work, our first main result is the introduction of a simple variant of the SCJ model that accounts for single-gene duplications, for which computing the directed distance from a trivial ancestral genome to a non-trivial descendant genome can be done in linear time. This is a somewhat surprising tractability result as some relatively similar problems are known to be intractable, such as the (1, 2)-exemplar breakpoint distance [19]. The requirement of considering a trivial ancestral genome and of assuming unambiguous orthology relations is crucial toward our tractability result and is motivated by applications toward the small parsimony problem. Moreover it is relevant toward applications as recent progress in reconciliation algorithms make it realistic to assume that the gene content and orthology relations are known at all nodes of a given species phylogeny; we refer to [22, 23, 28] for a series of papers describing this approach and applying it on real data. From a theoretical point of view, it remains to be seen if these assumptions can be lifted, although this makes the problem very close to general breakpoint distance with duplicated genes, that has been shown to be intractable [29]. Generally, we believe it is worthwhile, both from a theoretical point of view and an applied point of view, to push the tractability boundaries of the SCJ models toward augmented models of evolution (here accounting for duplications).

Our other results deal with the median genome; we show an intriguing tractability boundary between the directed median problem and the rooted median problem, while in the SCJ model with no duplicated genes, both problems are equivalent and the median problem is tractable [12]. An interesting feature of our hardness proof is that it relies on two identical descendant genomes, showing a sharp tractability boundary between the directed pairwise distance problem and the rooted median of three genomes problem. Similarly to other SCJ-related median problems, our rooted median problem aims at selecting adjacencies among candidate adjacencies which are seen in a majority of the given input genomes; nevertheless the possibility of conflicting median adjacencies due to convergent evolution is at the heart of the intractability of the problem. A consequence of the hardness of the rooted median problem is that it likely implies the hardness of the Small Parsimony Problem in augmented SCJ model, when the considered species phylogeny is rooted. Again this contrasts with the classical SCJ model for which the small parsimony problem is tractable [12].

To address the intractability of the rooted median problem, we provide a simple Integer Linear Program that computes an optimal median. Without surprise, we observe that our ILP outputs a more reliable estimate of the median in case of lower rates of rearrangements. Moreover, we observe that despite having many more optimal solutions for higher rates of rearrangement, the distance of a random solution from the actual median does not deviate by much. This suggests that in practice, the rooted median problem in our model is relatively easy to solve.

Our work leaves several open questions. The most natural one asks if our model can be extended to include other kinds of duplications, other than single-gene duplications. It was shown in [20] that Whole-Chromosome Duplications can be handled, although it is much more complicated to compute the distance. It is then relevant to ask if an intermediate model accounting for a wider range of duplication mechanisms can lead to tractable distance problems. Accounting for gene duplication naturally leads to considering gene loss. So far our results assume all genomes have equal gene family content, which combined with the requirement of unambiguous orthology relations, imply that we do not consider gene losses. It is not difficult to model gene loss in our model, using cuts and joins to extract lost genes into single-gene circular chromosomes, the symmetric operation of a floating duplication. However, in preliminary experiments on real and simulated data (not shown), this leads to a dramatic increase of the distance, driven by gene losses. The question of modeling gene losses with SCJ was previously raised in [20] and is still largely open. Last, the question of counting or sampling optimal evolutionary scenarios, both between two genomes or in the median problem comes to mind. When two genomes are considered, it was shown in [16] that the exact number of SCJ scenarios can be computed in polynomial time through simple recurrences, that also lead to a sampling algorithm; for the median problem, it follows immediately from the algorithm described in [12] that optimal medians can be counted and sampled easily (actually there is a unique optimal median if *k* is odd). However, both techniques do not extend immediately to our model, especially because an adjacency multi-set does not have a unique realization as a gene order with duplicated genes. So counting and sampling optimal evolutionary scenarios in our model is an open question deserving further research.

## Supplementary information

**Additional file 1.** Proofs.

## Data Availability

Data analysed during the study was generated using the ZOMBI genome simulator. https://github.com/cchauve/SCJ-with-SGD.

## Footnotes

1 https://github.com/cchauve/SCJ-with-SGD.
