# Supplementary material for: The distance and median problems in the single-cut-or-join model with single-gene duplications
Source: Algorithms Mol Biol. 2020 May 4;15:8. doi: 10.1186/s13015-020-00169-y (PMC7197181; doi:10.1186/s13015-020-00169-y)
Supplement: Supplementary file 1 — Additional file 1. Proofs. [file 13015_2020_169_MOESM1_ESM.pdf]

Acknowledgements
Most computations were done on the Cedar system of ComputeCanada through a resource allocation to CC.

Figures
Tables
Additional Files
Additional file 1 — Proofs
Additional file 1 contains the proof omitted in the main text.

Additional File 1

Proof of Theorem 1. First, we state an immediate result related to the reduction process:

Lemma 7 $d_{DSCJ}(A, D) = d_{DSCJ}(A, r(D)) + t(D)$.

As a consequence, we assume from now on that D has been reduced and does not contain any tandem array or any extra copy of a non-trivial family that is in a single-gene circular chromosome, and we prove that

$$d_{\mathrm{DSCJ}}(A, D) = |A - D| + |D - A| + 2\delta(A, r(D)).$$

For the sake of exposition, from now we denote $\delta(A, r(D))$ by d.

First, we show that $d_{\mathrm{DSCJ}}(A, D) \geq |A - D| + |D - A| + 2d$. To obtain D from A, we need exactly d gene duplications. Each duplication of a gene g will create the adjacency $g_h g_t$, regardless of the type of the duplication or the timing of the duplication event. Therefore, d adjacencies of the type $g_h g_t$ will have to be cut, as D is reduced and has no adjacency of this type. In addition, any adjacency in $A - D$ and $D - A$ defines an unavoidable cut or join respectively. Therefore, we can not transform A into D with less than $|A - D| + |D - A| + 2d$ operations.

Now, we show that $d_{\mathrm{DSCJ}}(A, D) \leq |A - D| + |D - A| + 2d$, by induction on d. For the base case $d = 0$, the result follows immediately as both genomes are trivial and $d_{\mathrm{DSCJ}}(A, D) = d_{\mathrm{SCJ}}(A, D)$.

We now assume that $d > 0$, and pick a gene g with one copy in A and more than one copy in D. Depending on how the adjacencies of g are conserved or not in D, we have a few different subcases to consider. However, in each subcase the general strategy remains the same, as follows. We build a genome A_2 from A by applying one duplication (FD or TD) and also relabeling the original copy g as g', creating an adjacency $g_h g_t$ in the case of an FD or $g'_h g_t$ in the case of a TD. Then we build a genome D_2 from D by also relabeling one copy of g to g', thus creating a new trivial gene family and an instance of the d-SCJ-TD-FD problem with exactly $d-1$ duplicated gene copies. We can apply the induction hypothesis, leading to the inequality

$$d_{\mathrm{DSCJ}}(A_2, D_2) \leq |A_2 - D_2| + |D_2 - A_2| + 2(d - 1).$$

Also, as D and D_2 are identical but for the relabeling of g, there is a scenario from A to D, going from A to A_2 and then to D, resulting in the upper bound

$$d_{\mathrm{DSCJ}}(A, D) \leq d_{\mathrm{DSCJ}}(A, A_2) + d_{\mathrm{DSCJ}}(A_2, D_2) = 1 + d_{\mathrm{DSCJ}}(A_2, D_2).$$

We will then show that we can build A_2 and D_2 in a way that they satisfy

$$|A - D| + |D - A| = |A_2 - D_2| + |D_2 - A_2| - 1,$$

where the -1 term is due to the extra $g_h g_t$ adjacency on A_2 created with the duplication. Together with the above inequalities this will lead to

$$d_{\text{DSCJ}}(A, D) \leq 1 + d_{\text{DSCJ}}(A_2, D_2) \leq |A - D| + |D - A| + 2d$$

and the result follows. To show that we can build A_2 and D_2 that satisfy the above conditions, we will consider three subcases.

Case (i): Assume that g is not a telomere (and so there are two adjacencies involving g in A, say xg_t and $g_h y$) and there is a copy of g in D whose extremities form also adjacencies xg_t and $g_h y$. We say that the context of g is *strongly conserved* between A and D. Note that x and y do not need to belong to trivial gene families and there might be several copies of x, y, g in D that conserve the context of g in A.

In this case, we build A_2 by applying an FD to create an extra copy of g and relabel the original copy of g in A as g'; we also relabel g' an arbitrary copy of g in D that has the same context than g in A, to obtain D_2 (see Fig. 6. Comparing the adjacency sets of A and D with A_2 and D_2, we can see that from A to A_2 two adjacencies where renamed from xg_t and $g_h y$ to xg'_t and $g'_h y$, and exactly the same change happened from D to D_2. Also, the adjacency $g_h g_t$ was added in A_2. As a result, $A_2 = A - \{xg_t, g_h y\} + \{xg'_t, g'_h y, g_h g_t\}$. Similarly, $D_2 = D - \{xg_t, g_h y\} + \{xg'_t, g'_h y\}$. Therefore, we have that $|A - D| + |D - A| = |A_2 - D_2| + |D_2 - A_2| - 1$. Note that this relabeling only works if we introduce a an extra copy of g in A with an FD here; if instead we introduce it with a TD, it would not be possible to get adjacencies xg'_t and $g'_h y$ in D_2, as the copy of g involved in both adjacencies would be different.

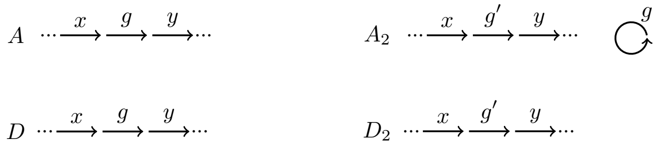

Figure 6 The context of g is strongly conserved between A and D (Case (i)).

Case (ii): Assume that g is not a telomere in A, its context is not strongly conserved between A and D, but both adjacencies involving g, xg_t and $g_h y$, are present in D *on different copies* of g. We say that the context of g is *weakly conserved* between A and D. Again x and y need not to be trivial gene families and there might be several occurrences of adjacencies xg_t and $g_h y$ in D.

In this case, we build A_2 by applying a TD on g, relabeling the gene g that has the adjacency xg_t as a new gene g' in both A_2 and D_2, as shown on Fig. 7. Comparing the adjacency sets of A and A_2, we notice that the adjacency xg_t changes to xg'_t, and $g_h g_t$ is added. Thus, $A_2 = A - \{xg_t\} + \{xg'_t, g_h g_t\}$. From D to D_2 we also have the same change, and possibly one more, depending if g'_h is a telomere in D (no change) or if g'_h has an adjacency $g'_h w$. In the former case, $D_2 = D - \{xg_t\} + \{xg'_t\}$.

Otherwise, $D_2 = D - \{xg_t, g_hw\} + \{xg'_t, g'_hw\}$. In either case, the possible adjacency g'_hw does not exist in A or A_2. Consequently, the equality $|A - D| + |D - A| = |A_2 - D_2| + |D_2 - A_2| - 1$ holds.

Note also that in this case an FD would not be optimal, because it would force the labeling of the adjacency g_hy to g'_hy, and since the adjacency g_hy on D cannot have the label g'_hy, this would force an extra pair of SCJ operations.

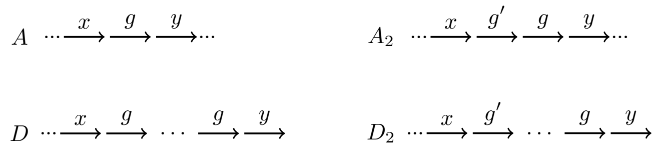

Figure 7 The context of g is weakly conserved between A and D (Case (ii)).

Case (iii) : We assume now that the context of g in A is neither strongly nor weakly conserved, and so at most one adjacency of g in A is also present in D.

This case is similar to case (i), if we assume that either xg_t or g_hy, are present in D, or neither. In the same way, we apply an FD on g, labeling the original copy as g', as shown in Fig. 8. On D, we pick a gene g that has an adjacency xg_t or g_hy if any or, if no adjacency involving g is conserved in D, we pick an arbitrary g, and relabel it as g'.

Now, any adjacencies that were conserved between A and D will remain conserved between A_2 and D_2, and no new conserved adjacencies have been created. Since, as before, A_2 has a new g_hg_t adjacency, the equality $|A - D| + |D - A| = |A_2 - D_2| + |D_2 - A_2| - 1$ holds.

These three cases cover all possible configurations for g, so the theorem is proved. □

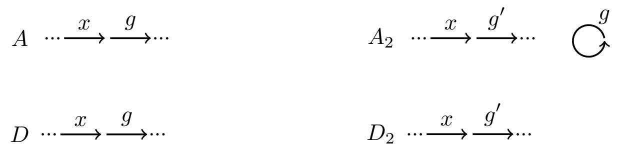

Figure 8 At most one adjacency of g is conserved (Case (iii)).

Proof of Corollary 2. From Theorem 1, we can easily transform the SCJ-TD-FD distance formula into

$$d_{\mathrm{DSCJ}}(A, B) = |A - r(D)| + |r(D) - A| + 2\delta(A, D) - t(D). \tag{13}$$

Indeed we remind that the original pairwise distance formula (eq. (3)) is

$$d_{\mathrm{DSCJ}}(A, D) = |A - r(D)| + |r(D) - A| + 2\delta(A, r(D)) + t(D).$$

Consider the difference in the number of genes from D to $r(D)$. Each time we remove a $g_h g_t$ observed duplication from D while reducing it, it corresponds to removing a copy of g from D. Thus D has $t(D)$ more genes than $r(D)$, so that $2\delta(A, D) = 2\delta(A, r(D)) + 2t(D)$. This implies $2\delta(A, D) - t(D) = 2\delta(A, r(D)) + t(D)$.

However, it is easier to express the distance without the reduced genome terms. Hence, we eliminate the need for computing the reduced genomes by replacing $|A - r(D)|$ and $|r(D) - A|$ by suitable expressions as follows. We show that (1) $|A - r(D)| = |A - D| + \sum_{g \in \Gamma_A} \alpha_g$, and (2) $|r(D) - A| = |D - A| - t(D) + \sum_{g \in \Gamma_A} \alpha_g$. Substituting the terms in eq. (13) yields eq. (4).

(1) Consider first the difference between $A - r(D)$ and $A - D$. Suppose that adjacency xy is in $A - D$ ($xy \in A - D$) but $xy \notin A - r(D)$. Then $xy \in r(D)$ but $xy \notin D$, which is not possible. Thus the difference can only be due to some $xy \in A - r(D)$ such that $xy \notin A - D$. This means that $xy \notin r(D)$ and $xy \in D$, which only happens when $xy = g_h g_t$ for some gene g. As we have $xy = g_h g_t \in A \cap D$ and $g_h g_t \notin r(D)$, we also have $\alpha_g = 1$, by definition. Since only one such adjacency is possible for each gene g (because A is trivial), $A - r(D)$ and $A - D$ differ only by adjacencies on genes for which $\alpha_g = 1$. We have shown that $|A - r(D)| = |A - D| + \sum_{g \in \Gamma_A} \alpha_g$.

(2) Now consider the difference between $r(D) - A$ and $D - A$. Note that there are $t(D)$ adjacencies in D not in $r(D)$, all observed duplications of the type $g_h g_t$. Let $g \in \Gamma_A$. If $g_h g_t \notin A$, then all of the $t(g)$ observed duplications in g are counted in $D - A$ but not in $r(D) - A$. This is also true when $g_h g_t \in A$ and $g_h g_t \in r(D)$. In these cases, $\alpha_g = 0$. However when $g_h g_t \in A \cap D$ but $g_h g_t \notin r(D)$, there are $t(g) - 1$ of the $g_h g_t$ adjacencies counted in $D - A$ not counted in $r(D) - A$ (this is because exactly one $g_h g_t$ adjacency of v can be matched with the $g_h g_t$ adjacency in A, and $r(D)$ has no such adjacency). This case occurs precisely when $\alpha_g = 1$. This shows that $|r(D) - A| = |D - A| - \sum_{g \in \Gamma_A} (t(g) - \alpha_g) = |D - A| - t(D) + \sum_{g \in \Gamma_A} \alpha_g$. □

Proof of Theorem 5. We show that finding the optimal gene order for M is NP-hard even for $k = 2$, by reduction from the 2P2N-3SAT problem [?][2]. In 2P2N-3SAT, we are given n variables $x_1, \ldots, x_n$ and m clauses $C_1, \ldots, C_m$, each containing exactly 3 literals. Each x_i variable appears as a positive literal in exactly 2 clauses, and as a negative literal in exactly 2 clauses. Note that since each variable occurs in exactly 4 clauses and each clause has 3 literals, $m = 4n/3$. An example of a 2P2N-3SAT instance is shown in Figure 9 (top left).

We now describe how we transform the x_i variables and C_j clauses into an instance of the rooted median. The genes of M are

$$\Gamma = \{g_1^+, \gamma_1^+, g_1^-, \gamma_1^-, \ldots, g_n^+, \gamma_n^+, g_n^-, \gamma_n^-, c_1, \ldots, c_m, \alpha_1, \ldots, \alpha_{2n-m}\}$$

The genes $g_i^+, \gamma_i^+, g_i^-, \gamma_i^-$ correspond to the x_i variable, and c_j to the clause C_j. The purpose of the $2n - m = 2n/3$ special α_i genes will become apparent later.

To simplify matters, every adjacency in our reduction is between the tails of two genes. Hence, the heads of each gene of A, D_1 and D_2 are telomeres (linear chromosomes extremities), so that all chromosomes are linear and have at most 2 genes.

[2]This problem is sometimes called the (3,B2)-SAT problem, where B2 indicates that the literals are balanced with two occurrences each.

From now, we will omit the t subscript from the extremities for these adjacencies, with the understanding that every adjacency is between tails; for instance, we may write $g_i^+\gamma_i^+$ for the adjacency $g_{i,t}^+\gamma_{i,t}^+$.

We can now describe A, D_1 and D_2. The genes of A are $g'_1, \gamma'_1, \ldots, g'_n, \gamma'_n$, $c'_1, \ldots, c'_m, \alpha'_1, \ldots, \alpha'_{2n-m}$. The genes g_i^+ and g_i^- (resp. γ_i^+ and γ_i^-) are duplicates of g'_i (resp. γ'_i), and there are no other duplications in M compared to A. Formally, for each $i \in [n]$, put $a(g_i^+) = a(g_i^-) = g'_i$, $a(\gamma_i^+) = a(\gamma_i^-) = \gamma'_i$ and for each $j \in [m]$, put $a(c_j) = c'_j$. Finally, for each $i \in [2n-m]$, put $a(\alpha_i) = \alpha'_i$. The adjacencies of A are $\{g'_i\gamma'_i : i \in [n]\}$.

The genomes D_1 and D_2 are identical, i.e. they contain the same set of genes and of adjacencies. We simply describe the set of adjacencies of D_1 and D_2 with the understanding that if an extremity, say x, appears in two adjacencies xy and xz, then the two x are the tails of two distinct copies of the same gene on two distinct chromosomes. The adjacencies of D_1 and D_2 are described as follows.

- For each $i \in [n]$, add to D_1 and D_2 the adjacencies $g_i^+\gamma_i^+$ and $g_i^-\gamma_i^-$.
- For each $i \in [n]$, let C_{j_1}, C_{j_2} be the two clauses in which x_i occurs positively and let C_{k_1}, C_{k_2} be the two clauses in which x_i occurs negatively. Add to D_1 and D_2 the adjacencies $g_i^+c_{j_1}$ and $\gamma_i^+c_{j_2}$. Similarly, add to D_1 and D_2 the adjacencies $g_i^-c_{k_1}$ and $\gamma_i^-c_{k_2}$[3].
- Finally, for each $i \in [n]$ and each $j \in [2n-m]$, add to D_1 and D_2 the adjacencies $g_i^+\alpha_j, g_i^-\alpha_j, \gamma_i^+\alpha_j$ and $\gamma_i^-\alpha_j$.

This completes our construction.

The intuition behind our hardness proof is that for each $i \in [n]$, we need to pick one of $g_i^+\gamma_i^+$ or $g_i^-\gamma_i^-$ in M, as we will show. Simultaneously, we would like to include as many adjacencies which are in both D_1 and D_2. It will possible to choose the positive and negative adjacencies *and* match all the c_j and α_j if and only if the 2P2N-3SAT instance is satisfiable.

It will be useful to think of D_1 (and D_2) as the set of adjacencies which are allowed to belong to M, as stated in the following.

Lemma 8 *Let a be an adjacency in M, such that $a \notin D_1$ (equivalently, $a \notin D_2$). Then $M - \{a\}$ achieves a smaller total distance to A, D_1 and D_2 than M.*

Proof By cutting a, we increase the distance to A by at most 1, but decrease the distance to D_1 and D_2 by 1 each. This is because $|(M - \{a\}) - D_1| + |D_1 - (M - \{a\})| = |M - D_1| - 1 + |D_1 - M|$, the value of $\delta(M, D_1)$ is unchanged and $t(D_1) = 0$ by assumption (and the same holds for D_2). Therefore removing a from M yields a better median genome. □

Therefore, we may assume that every adjacency of a median M belongs to D_1 and D_2. Note that this implies that M contains no observed duplications (with respect to A), as no such adjacency is in D_1 and D_2. Thus we will ignore the $t(M_a) = 0$ term in $d_{\mathrm{DSCJ}}(A, M_a)$ (eq. (13)), and we will not make a distinction between M_a and $r(M_a)$, as these are equal.

[3] Intuitively, these adjacencies represent using a literal to satisfy a specific clause. For instance, the adjacency $g_i^+c_{j_1}$ represents "setting x_i to true and satisfying C_{j_1}".

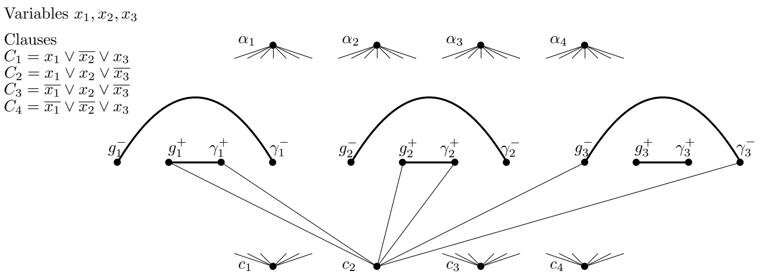

Figure 9 An example of a 2P2N-3SAT instance, with an illustration of the genes of M (only the gene tails are shown) and the adjacencies that are allowed by D_1 and D_2. The fat edges represent pairs of adjacencies of which at least one must be present according to Lemma 8.

Another property of M is that it must contain at least one "positive" or one "negative" adjacency for each $i \in [n]$.

Lemma 9 *For $i \in [n]$, M contains at least one of $g_i^+\gamma_i^+$ and $g_i^-\gamma_i^-$.*

Proof Suppose that for some i, M contains none of $g_i^+\gamma_i^+$ or $g_i^-\gamma_i^-$. Note that M does not contain $g_i^+\gamma_i^-$ nor $g_i^-\gamma_i^+$, by Lemma 8. This implies that $g_i'\gamma_i' \notin M_a$, as we have excluded all the four possibilities of having this adjacency in M_a.

Consider the median M' obtained from M by adding $g_i^+\gamma_i^+$, cutting the adjacencies that g_i^+ and γ_i^+ were contained in, if needed. If g_i^+ and γ_i^+ are both telomeres in M, then it is easy to check that $M' = M + g_i^+\gamma_i^+$ (M augmented by the adjacency $g_i^+\gamma_i^+$) attains a better distance than M since $g_i^+\gamma_i^+ \in D_1, D_2$ and $a(g_i^+)a(\gamma_i^+) = g_i'\gamma_i' \in A$ (this decreases the distance by 3).

Suppose that $g_i^+x \in M$ for some x, and that γ_i^+ is a telomere in M. By Lemma 8, g_i^+x is in both D_1 and D_2, which implies that $x = c_j$ or $x = \alpha_j$ for some j. This implies in turn that $a(g_i^+)a(x) \notin A$. We can argue that $M' = M - g_i^+x + g_i^+\gamma_i^+$ is better. To see this, observe that $|M' - D_1| = |M - D_1|$ and $|D_1 - M'| = |D_1 - M|$ (and the same with D_2). On the other hand, recalling that $g_i'\gamma_i' \notin M_a$, we have $|M_a' - A| = |M_a - A| - 1$ (because $a(g_i^+)a(x) \notin A$ and $a(g_i^+)a(\gamma_i^+) \in A$) and $|A - M_a'| = |A - M_a| - 1$ (because $a(g_i^+)a(\gamma_i^+) \in A$). We have thus decreased the distance by 2. The same argument applies if g_i^+ is a telomere but γ_i^+ is not.

Finally, suppose that g_i^+x and γ_i^+y are adjacencies of M. As we argued above, $a(g_i^+)a(x) \notin A$ and $a(\gamma_i^+)a(y) \notin A$. Letting $M' = M - g_i^+x - \gamma_i^+y + g_i^+\gamma_i^+$, we find that $|M' - D_1| = |M - D_1|$ and $|D_1 - M'| = |D_1 - M| + 1$. As the same holds with D_2, we have increased the distance to D_1 and D_2 by 2. On the other hand, $|A - M_a'| = |A - M_a| - 1$ and $|M_a' - A| = |M_a - A| - 2$. To sum up, the total distance decreases by 1. □

We now formally prove the hardness of computing the rooted SCJ-TD-FD median.

Theorem 5 Let $x_1, \ldots, x_n$ and $C_1, \ldots, C_m$ be a 2P2N-3SAT-instance, and let A, D_1, D_2 and the genes Γ of M be the corresponding instance of the r-SCJ-TD-FD median genome problem. We will show that the given 2P2N-3SAT instance is

satisfiable if and only if there exists a median genome M satisfying

$$d_{\text{DSCJ}}(A, M_a) + d_{\text{DSCJ}}(M, D_1) + d_{\text{DSCJ}}(M, D_2) \leq 2|D_1| - 2n + 4\delta(M, D_1)$$

($\Rightarrow$) Suppose that the 2P2N-3SAT can be satisfied by an assignment of the x_i variables to true or false. Construct a median genome using the following steps.

1 For each $i \in [n]$, if x_i is set to true, then add $g_i^- \gamma_i^-$ to M, and if instead x_i is set to false, add $g_i^+ \gamma_i^+$ to M.

2 Then, add to M these adjacencies in an algorithmic fashion: for each $j = 1, 2, \ldots, m$, consider clause C_j and let x_i be any variable satisfying C_j.
  - If x_i is set to true, then note that g_i^+ and γ_i^+ have not been matched in Step 1. Add $g_i^+ c_j$ to M if g_i^+ is not part of an adjacency of M yet, or add $\gamma_i^+ c_j$ to M otherwise.
  - If instead x_i is set to false, then g_i^- and γ_i^- have not been matched in Step 1. Add $g_i^- c_j$ if g_i^- is not part of an adjacency in M yet, or add $\gamma_i^- c_j$ to M otherwise.

  Note that since each x_i can satisfy at most two clauses, it will always be possible to find an extremity to match c_j with.

3 Finally, observe that so far each of the g_i^+, g_i^-, γ_i^+ and γ_i^- extremities are in an adjacency M, except $4n - 2n - m = 2n - m$ of them. Associate each such extremity g with a distinct α_j extremity arbitrarily, and add each $g\alpha_j$ to M, noting that there are just enough α_j genes to do so.

Note that M contains $n+m+2n-m = 3n$ adjacencies in total, exactly n of which correspond to an adjacency of A (those included in Step 1). Also, every adjacency of M occurs in both D_1 and D_2. We have

$$\begin{aligned} d_{\text{DSCJ}}(A, M_a) &= |A - M_a| + |M_a - A| + 2\delta(A, M_a) - t(M_a) \\ &= 0 + 2n + 2n - 0 = 4n \end{aligned}$$

As for D_1 and D_2,

$$\begin{aligned} d_{\text{DSCJ}}(M, D_1) = d_{\text{DSCJ}}(M, D_2) &= |D_1 - M| + |M - D_1| + 2\delta(M, D_1) \\ &= |D_1| - 3n + 0 + 2\delta(M, D_1) \end{aligned}$$

Therefore the total distance is $4n + 2(|D_1| - 3n + 2\delta(M, D_1)) = 2|D_1| - 2n + 4\delta(M, D_1)$, as we predicted.

($\Leftarrow$) Suppose that there exists a median genome M of total distance at most $2|D_1| - 2n + 4\delta(M, D_1)$. By Lemma 8, we may assume that every adjacency of M is present in both D_1 and D_2.

With the next two claims, we will prove that M has exactly $3n$ adjacencies, of which exactly n are adjacencies corresponding to those in A.

Claim 2 *$|M| \leq 3n$, and $|M| = 3n$ only if every c_j and α_j extremity is in some adjacency of M.*

Proof Call an extremity e of a gene in Γ *matchable* if there exists an adjacency of D_1 that contains e. By Lemma 8, the adjacencies of M only contain matchable extremities. The g_i^+, g_i^-, γ_i^+ and γ_i^- extremities account for $4n$ matchable extremities. The c_j genes account for m matchable extremities and the α_j genes for $2n-m$ matchable extremities . Thus there are $4n+m+2n-m = 6n$ matchable extremities. Because an adjacency contains 2 extremities, there can be at most $3n$ adjacencies in M. The second part of the claim follows from the fact that we have to assume that every c_j and α_j is matched to attain this bound. □

For the rest of the proof, denote by q the number of distinct adjacencies $ab \in A$ for which there exists $xy \in M$ such that $a(x)a(y) = ab$.

Claim 3 *$|M| = 3n$ and $q = n$.*

Proof By the definition of q, we have $|A - M_a| = n - q$ and $|M_a - A| = |M| - q$. It follows that

$$\begin{aligned} d_{\mathrm{DSCJ}}(A, M_a) &= |A - M_a| + |M_a - A| + 2\delta(A, M_a) - t(M_a) \\ &= n - q + |M| - q + 2n - 0 \\ &= |M| + 3n - 2q \end{aligned}$$

Using Lemma 8, we also have $d_{\mathrm{DSCJ}}(M, D_1) = |M - D_1| + |D_1 - M| + 2\delta(M, D_1) = 0 + |D_1| - |M| + 2\delta(M, D_1)$. Thus the sum of the 3 distances is

$$|M| + 3n - 2q + 2|D_1| - 2|M| + 4\delta(M, D_1) \leq 2|D_1| - 2n + 4\delta(M, D_1)$$

(this inequality is due to our initial assumption on the total distance of M). After simplifying, this gives $5n \leq |M| + 2q$. By Claim 2, $|M| \leq 3n$ and because A has n adjacencies, $q \leq n$. Hence, this inequality is only possible if $|M| = 3n$ and $q = n$. □

Because $q = n$, Claim 3 implies that for each $i \in [n]$, (at least) one of $g_i^+\gamma_i^+$ and $g_i^-\gamma_i^-$ is in M. This lets us define as assignment for our 2P2N-3SAT instance: for each $i \in [n]$, set x_i to *true* if $g_i^-\gamma_i^-$ is in M, and otherwise set x_i to *false*. We claim this this assignment satisfies every clause.

To see this, let C_j be a clause and let c_j be its corresponding extremity in M. By Claim 3, every extremity that is part of some adjacency in D_1 must be part of an adjacency in M, including c_j. Thus there is some e such that $c_j e \in M$. By Lemma 8, the adjacency $c_j e$ must also be in D_1, and by construction either (1) $e \in \{g_i^+, \gamma_i^+\}$ for some x_i that occurs positively in C_j, or (2) $e \in \{g_i^-, \gamma_i^-\}$ for some x_i that occurs negatively in C_j. Suppose that case (1) applies. Then $c_j g_i^+$ or $c_j \gamma_i^+$ being in M means that $g_i^+\gamma_i^+ \notin M$, implying in turn that $g_i^-\gamma_i^-$ is in M. In this situation, we have set x_i to *true* and we satisfy C_j. Suppose instead that case (2) applies. Then $g_i^-\gamma_i^- \notin M$, in which case we have set x_i to false and satisfy C_j. As the argument applies to any clause C_j, this concludes the proof. □

Proof of Claim (1). By eq. (4), we know that

$$d_{\text{DSCJ}}(A, M_a) = |A - M_a| + |M_a - A| + 2\delta(A, M_a) - 2t(M_a) + 2\sum_{g\in\Gamma_A} \alpha_{g,AM_a}$$

$$d_{\text{DSCJ}}(M, D_i) = |M - D_i| + |D_i - M| + 2\delta(M, D_i) - 2t(D_i) + 2\sum_{g\in\Gamma_M} \alpha_{g,MD_i}$$

where Γ_A and Γ_M are the set of genes in the gene orders of A and M, respectively, and so also the genes alphabets for M and the D_is. Variables α_{g,AM_a} and α_{g,MD_i} are defined as $\alpha_{g,uv}$ above.

For any two adjacency sets X and Y, we use the identity $|X - Y| + |Y - X| = |X| + |Y| - 2|X \cap Y|$ to obtain

$$d_{\text{DSCJ}}(A, M_a) = |A| + |M_a| - 2|A \cap M_a| + 2\delta(A, M_a) - 2t(M_a) + 2\sum_{g\in\Gamma_A} \alpha_{g,AM_a},$$

$$d_{\text{DSCJ}}(M, D_i) = |M| + |D_i| - 2|M \cap D_i| + 2\delta(M, D_i) - 2t(D_i) + 2\sum_{g\in\Gamma_M} \alpha_{g,MD_i}.$$

This eliminates the need to count the actual number of cut and join events along every branch. Instead, it suffices to compute the common adjacencies in the parent and child genomes (using the terms $|A\cap M_a|$ and $|M\cap D_i|$) for each branch (A, M_a) and (M, D_i).

For a median M, let $s(M) = d_{\text{DSCJ}}(A, M_a) + \sum_{i=1}^{k} d_{\text{DSCJ}}(M, D_i)$ be the *score* of M. It follows easily from above that

$$\begin{aligned} s(M) = &\left[|A| + 2\delta(A, M_a) + \sum_{i=1}^{k} (|D_i| + 2\delta(M, D_i))\right] \\ &- \left[\sum_{i=1}^{k} \left(2|M \cap D_i| + 2t(D_i) - 2\sum_{g\in\Gamma_M} \alpha_{g,MD_i}\right)\right. \\ &\left. + 2|A \cap M_a| + 2t(M_a) - 2\sum_{g\in\Gamma_A} \alpha_{g,AM_a} - (k+1)|M|\right] \end{aligned}$$

Let $N = |A| + 2\delta(A, M_a) + \sum_{i=1}^{k} \left(|D_i| + 2\delta(M, D_i) + 2t(D_i)\right)$. Given that N depends only on A and D_i and not on M, it is constant (note that $\delta(A, M_a)$ and $\delta(M, D_i)$ are constant as the gene content of M is an input to the problem). Thus in order to minimize the score $s(M)$, we only need to maximize the term:

$$\sum_{i=1}^{k} \left(2|M \cap D_i| - 2\sum_{g\in\Gamma_M} \alpha_{g,MD_i}\right) + 2|A\cap M_a| + 2t(M_a) - 2\sum_{g\in\Gamma_A} \alpha_{g,AM_a} - (k+1)|M|$$

which is negated in $s(M)$, as required in eq. (5). □

Proof of Lemma (6). To prove this lemma, we start with a median containing a non-candidate adjacency. For odd values of k, we prove that removing the non-candidate adjacency results in another median of the same cost whereas for even k, it is shown that the resultant median (on removing the non-candidate adjacency) is better. We temporarily ignore the influence of reduced genomes for this proof.

Consider an adjacency xy that is not a candidate. Recall that since xy is not a candidate it is present in at most $\lfloor \frac{k+1}{2} \rfloor$ genomes from $\{A, D_1, ..., D_k\}$. Assume that M is a median genome and xy is present in M. Further, assume that M is optimal. Thus, the sum of the distances $d_{\text{DSCJ}}(A, M_a) + \sum_{i=1}^{k} d_{\text{DSCJ}}(M, D_i)$ should be the least over all medians. Let M' be the genome obtained by removing xy from M.

Let $D_{xy} \subseteq \{D_1, ..., D_k\}$ be the set of descendant genomes that contain xy, and let $\overline{D_{xy}}$ be the set of those that do not. For any $D_i \in D_{xy}$, the adjacency need not be cut along (M, D_i), however it has to be added along (M', D_i), introducing an extra cost of 1 to the total distance. Thus, $d_{\text{DSCJ}}(M, D_i) = d_{\text{DSCJ}}(M', D_i) - 1$, for all $D_i \in D_{xy}$. On the other hand, if $D_i \notin D_{xy}$, then it does not contain xy. Consequently, for all such D_i, the adjacency has to be cut along (M, D_i) but not along (M', D_i) (since M' does not contain it in the first place). Thus, for all $D_i \notin D_{xy}$, $d_{\text{DSCJ}}(M, D_i) = d_{\text{DSCJ}}(M', D_i) + 1$.

Further if A contains $a(x)a(y)$, it need not be cut along (A, M_a) but may need to be cut along (A, M'_a) thereby introducing a possible extra cost of 1 (note here the possibility that some $x^*y^* \in M$ distinct from xy such that $a(x^*)a(y^*) = a(x)a(y)$). Thus, $d_{\text{DSCJ}}(A, M_a) \geq d_{\text{DSCJ}}(A, M'_a) - 1$. If instead, A does not contain xy then it has to be joined along (A, M) and not along (A, M'_a). Unlike the previous case, the cost of the join is unavoidable. Hence, $d_{\text{DSCJ}}(A, M_a) = d_{\text{DSCJ}}(A, M'_a) + 1$.

Case 1: A contains xy. Then $|D_{xy}| \leq \lfloor \frac{k+1}{2} \rfloor - 1$.

$$
\begin{aligned}
&d_{\text{DSCJ}}(A, M_a) \geq d_{\text{DSCJ}}(A, M'_a) - 1 && \\
&d_{\text{DSCJ}}(M, D_i) = d_{\text{DSCJ}}(M', D_i) - 1 && \forall D_i \in D_{xy} \\
&d_{\text{DSCJ}}(M, D_i) = d_{\text{DSCJ}}(M', D_i) + 1 && \forall D_i \notin D_{xy}
\end{aligned}
$$

Summing over all the input genomes, we get

$$
\begin{aligned}
d_{\text{DSCJ}}(A, M_a) + \sum_{D_i \in D_{xy}} d_{\text{DSCJ}}(M, D_i) \geq d_{\text{DSCJ}}(A, M'_a) + \sum_{D_i \in D_{xy}} d_{\text{DSCJ}}(M', D_i) \\
+ |\overline{D_{xy}}| - (|D_{xy}| + 1)
\end{aligned}
$$

We know that $|D_{xy}| + 1 \leq \lfloor \frac{k+1}{2} \rfloor$. If k is even, $|\overline{D_{xy}}| > |D_{xy}| + 1$. Hence,

$$
d_{\text{DSCJ}}(A, M_a) + \sum_{D_i \in D_{xy}} d_{\text{DSCJ}}(M, D_i) > d_{\text{DSCJ}}(A, M'_a) + \sum_{D_i \in D_{xy}} d_{\text{DSCJ}}(M', D_i)
$$

Thus, the cost of M' is better than that of the optimal median M and we have a contradiction. If k is odd, then $|\overline{D_{xy}}| = |D_{xy}| + 1$ and hence both M and M' incur the same overall cost. In other words, the removal of a non-candidate adjacency does not increase the cost of the optimal median. Thus, iteratively

removing all such adjacencies will yield an optimal median that consists solely of candidate adjacencies.

Case 2: A does not contain xy. Then $|D_{xy}| \leq \left\lfloor \frac{k+1}{2} \right\rfloor$.

$$\begin{aligned} d_{\text{DSCJ}}(A, M) &= d_{\text{DSCJ}}(A, M') + 1 & \\ d_{\text{DSCJ}}(M, D_i) &= d_{\text{DSCJ}}(M', D_i) - 1 & \forall D_i \in D_{xy} \\ d_{\text{DSCJ}}(M, D_i) &= d_{\text{DSCJ}}(M', D_i) + 1 & \forall D_i \notin D_{xy} \end{aligned}$$

The analysis in this case is similar to Case 1. On adding all the equations and using $|D_{xy}| \leq \left\lfloor \frac{k+1}{2} \right\rfloor$, once again we reach a contradiction when k is even. When k is odd, both M and M' yield the same overall distance. Thus, we can still obtain the optimal median by iteratively removing non-candidate adjacencies.

Thus, when k is odd, there exists at least one optimal median consisting only of candidate adjacencies. However, when k is even, the optimal median must consist only of candidate adjacencies. □
